# Antitrypanosomal Effect of Hydromethanolic Extract of *Solanum anguivi* Lam on Field Isolates of Trypanosoma congolense Infected Mice

**DOI:** 10.1155/2021/1239379

**Published:** 2021-12-29

**Authors:** Debela Abdeta, Solomon Mequanente Abay, Mirutse Giday, Nigatu Kebede, Getechew Terefe

**Affiliations:** ^1^School of Veterinary Medicine, Wollega University, Nekemte, Ethiopia; ^2^School of Pharmacy, Addis Ababa University, Addis Ababa, Ethiopia; ^3^Akililu Lama Institutes of Pathobiology, Addis Ababa University, Addis Ababa, Ethiopia; ^4^Department of Parasitology, Addis Ababa University, Bishoftu, Ethiopia

## Abstract

**Introduction:**

Trypanosomiasis is one of the world's most serious infectious diseases caused by Trypanosoma parasites. Concern about resistance to conventional antitrypanosomal drugs, mosquito vector resistance to existing insecticide side effects of existing antitrypanosomal drugs justifies the urgent need for more effective, tolerable, and affordable drugs.

**Objective:**

The present study is aimed at determining the *in vivo* antitrypanosomal effect of the hydromethanolic extracts of *Solanum anguivi* fruit extracts against the field isolates of *T. congolense*.

**Methods:**

The 80% methanol extracts of *S. anguivi* fruits were prepared by cold maceration technique. *In vivo* curative tests were done to check the effect of plant extract against *T. congolense* in Swiss albino mice. Plant extracts were administered at doses of 100, 200, and 400 mg/kg/body weight. Acute toxicity of the extracts at 2000 mg/kg was performed according to OECD guidelines. Data obtained from the experiment were analyzed using one-way ANOVA followed by Tukey test.

**Results:**

This study indicated that extract did not exhibit any sign of acute toxicity up to 2000 mg/kg/body weight. In curative test, extracts reduced parasitemia, preventing the drop in packed cell volume and body weight significantly (*p* < 0.05), compared to control. Groups provided with the extract before infection got prolonged incubation period with chemoprophylactic effect at the doses of 100, 200, and 400 mg/kg. Phytochemical analysis showed presence of flavonoids, steroids, triterpens, saponins, glycosides, tannins, and alkaloids.

**Conclusion:**

The extract showed promising curative. Further effort is required to isolate and purify specific compounds responsible for antitrypanosomal activity of studied plant.

## 1. Introduction

Trypanosomes are protozoan parasite affecting both human and livestock. It is mainly found in Tropical Africa, Latin America, and Asia. It produces serious disease in human being such as sleeping sickness caused by *T.b.rodensia* and *T.b.gambesie* in Africa and Chagas disease caused by *T*. *cruzi* causes in Americas. In endemic areas, the other species of Trypanosoma affect animals and produce enormous economic impact [1]. African animal trypanosomiasis (AAT) is the most common diseases of covering 37 sub-Saharan countries located between latitudes 14°N and 29°S and about 9 million km^2^ land area [2]. This highly fatal protozoan disease is virulent and inoculable but not contagious (except dourine, a venereal trypanosomiasis of equines). African animal trypanosomiasis is responsible for 3 million livestock and 55,000 people death annually in agriculture and mixed farming system environments thus making it an important priority for the agricultural sector and biomedical and public agencies [3].

Current trypanosomiasis control relies on trypanocidal drugs, use of trypanotolerant cattle breeds, and controls of the vector, namely, the tsetse fly. None of these methods have the full potential to work in the long-term control of the disease. Most heavily relied on are the trypanocidal drugs, and this has led to an increasing problem of resistance in the target organisms [4]. Therefore, the search for new chemical entities that should be effective against all species of trypanosomes and safe and affordable for disease-endemic countries is the best choice left option to fight against the notorious impact of bovine trypanosomiasis on cattle productivity [5] and to reduce human loss due to human trypanosomiasis.

To control trypanosomiasis, researchers are seeking to find some alternative source of medications from natural sources due to the possible side effects of the existing drug [6]. Many herbal extracts like *Azadirachta indica*, *Acacia albica*, *Achyrocline* and *Indigofera oblongifolia* [7] *Khaya senegalensis*, *Piliostigma reticulatum*, *Securidaca longepedunculata*, *Ximenia Americana*, and *Artemisia abyssinica* [8–10].

These are found in plants which are potential sources of new drugs since they contain countless numbers of molecules that have pharmacological effects [11]. *Solanum anguivi* lam (local Afan Oromo name: “Hiddii seexanaa”) is a rare ethnomedicinal herb that belongs to the family Solanaceae and can be found throughout the nonarid parts of Africa. *S. anguivi* have been recognized to possess medicinal properties, and their use in traditional systems of medicine has been on record for a long time. It is highly polymorphic and variable in its plant structure, fruits, and leaf characters [12]. Although the plant is in use for the treatment of trypanosomiasis in Ethiopian, there is no laboratory-based evidence for the effectiveness and safety of the plant [13, 14]. Therefore, the study was carried out to determine the in vivo antitrypanosomal effect of hydromethanolic extract of *S. anguivi* fruit on mice experimentally infected with a field isolate of *T. congolense*.

## 2. Materials and Methods

### 2.1. Plant Collection and Authentication

In this study, *Solanum anguivi* fruits collected from Wayu Tuka district as of November 2015 were used as potential antitrypanosomial agent. Leaves with flower spacemen of the plant were collected, identified, and authenticated at Aklilu Lema Institute of Pathobiology, and the vouchers were deposited at the National Herbarium of Addis Abba University with number DA 02.

### 2.2. Preparation of Plant Extract

Dried powder of the plant material was macerated in 80% methanol in an Erlenmeyer flask for 72 hours at room temperature and periodically shaken with a mini orbital shaker. It was filtered twice with gauze and Whatman filter paper No. 1. Supernatants from the agitated material were separated from the undissolved portion. Using a rotary evaporator, methanol was removed from the filtrate. To remove water, the filtrate was lyophilized.

### 2.3. Ethical Approval

Ahead of starting data collection, ethical clearance was taken from research ethics committee (REC) of School of Veterinary Medicine, Wollega University, dated 15/09/2016 with minute no. SVM.RERC/004.

### 2.4. Experimental Animals

Swiss albino mice of either sex are weighing 30-35 g (age 10-12 weeks). They were purchased from Ambo University, bred in ALIPB's laboratory animal unit, and used for this study. A standard animal diet was fed to them, and they were watered frequently. They were fed with standard animal feed and watered *ad libitum* and maintained at room temperature of 23-25°C with a relative humidity of 60-65%. All procedures complied with the Guide for the Care and Use of Laboratory Animals [15].

### 2.5. Test Organism and Its Maintenance

*T. congolense* was obtained from Addis Ababa University Department of Veterinary Parasitology by infecting white albino mice intraperitoneally. Mice were screened for development of infection using buffy coat or Murray method [16].

### 2.6. Experimental Design

Thirty mice of male and female were randomly grouped in to five groups (I-V). All groups were injected with *T. congolense* (5∗10^5^ parasites/ml) infected blood. Groups I and II were administered Diminazene aceturate @3.335 mg/kg intraperitoneally, respectively, to serve as untreated and treated control whereas groups III-V administered at daily dose of 100, 200, and 400 mg/kg for consecutive 7 days from 10th day postparasite inoculation. Parasitemia and PCV were observed every 4 days for 21 days while body weight and rectal temperature were monitored every 2 days. Mean survival time was monitored for 6 weeks [17].

### 2.7. Parasitemia Determination

Parasite infected mice were checked for parasitemia every four days beginning on the tenth day after infection. The parasitemia of mice was monitored by microscopy at a magnification of 40x of blood obtained from the tail and examined by Herbert and Lumsden [18] formula using their rapid matching method. The method involves microscopically counting parasites per field in blood without diluting it. Logarithmic values of this count were obtained by matching with table of Herbert and Lumsden [18]. Monitoring of parasitemia was performed every four days to reduce stress on experimental animals until 21st days posttreatment initiation [19].

#### 2.7.1. Packed Cell Volume Determination

A microhaematocrit centrifuge and a microhaematocrit tube reader were used to determine PCV. PCV was performed every four days until 21st days posttreatment initiation [20, 21].

#### 2.7.2. Determination of Body Weight

A sensitive digital weighing balance was used to determine body weights of each mouse in each group on the day of parasite challenge, the day of treatment initiation, and every other day for 21 days [22].

#### 2.7.3. Rectal Temperature Determination

Digital thermometer (Mettle Toledo, Switzerland) was used to determine body temperature on the day of parasite inoculation, treatment commencement day, and every other day for 21 days [20].

#### 2.7.4. Mean Survival Time Determination

Survival rate of each mouse was recorded, and average life was determined for both treatment and control groups [8, 23].

### 2.8. Phytochemical Screening

Presence of plant secondary metabolite was assessed according to the methods described by different scholars.

Saponin, glycosides, tannins, and phlobatannins were carried out as described by Evans [24].

Terpenoid and steroids were carried out according to Briggs [25].

Tests for alkaloids and phenols were carried out according to [26, 27] where as flavonoid test was carried out according to Dermarderosian and Liberti's description [28]. The presence of anthroquinones was conducted as describe by [29] for free anthraquinone and combined anthroquinones.

### 2.9. Acute Toxicity of Crude Extract Determination

The median lethal doses (LD_50_) in mice were determined according to Lorke [30] method and organization for economic cooperation and development (OECD) guide line for testing of chemicals number 420 [31] on Swiss albino mice of female sex weighing 30-35 g and 10-12 weeks' age. The limit dose of 2000 mg/kg body weight was orally administered sequentially to five female mice and observed for 24 hours and then for additional 14 days. Toxicity signs like changes in physical appearance, behavioral changes and feeding activities, hair erection, lacrimation, reduction in motor, and other signs of acute toxicity and mortality were observed and recorded.

### 2.10. Statistical Analysis

Statistical Software Package for Social Science (SPSS) was used for data analysis. Data were presented as mean ± SEM. Significance was determined at 95% confidence level. Analysis of variance (one-way ANNOVA) was employed to test statistical difference with in all groups. *p* value less than 0.05 was considered statistically significant.

## 3. Results

### 3.1. Acute Toxicity

There was no death recorded 200 mg/kg body weight of the extract. Moreover, there were no gross behavioral changes and sign of toxicity during observation period which is monitored according to OECD 2001 guideline.

### 3.2. Effects of Hydromethanolic Extract *S. anguivi* Fruit Treatment on Parasitemia of *T. congolense* Infected Mice

**Figure 1:**
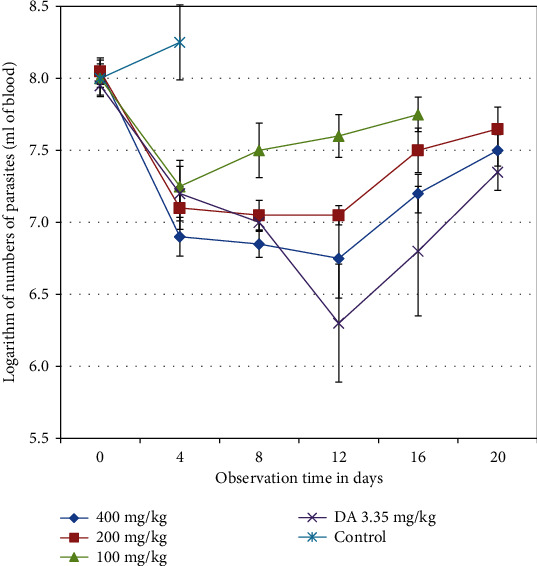
Effect of *S. anguivi* fruit extract treatment on *T. congolense* infected mice. Values are expressed as mean ± SEM; day 0: the 10th day after infected blood inoculation.

Preliminary screening for antitrypanosomal activity of hydomethanolic extract of *S. anguivi* revealed none of them completely cleared the Trypanosoma from the blood of infected mice. The pretreatment mean parasite count for all groups was around antilog 8.01 parasite/ml of blood. The changes observed in the level of parasitemia of infected treated mice were shown in Figure 1. Treatment with hydromethanolic extract of *S. anguivi* at 100 and 200 mg/kg showed a statically significant (*p* < 0.05) reduction in the level of parasitemia with in day 8 to day 12 posttreatment when compared with 400 mg/kg body weight.

Relapse was recorded for all treatment and positive control groups in which parasite started to be highly detected in the blood of infected mice. There is statistically significant association (*p* < 0.05) in parasitemia between those in 100 mg/kg of *S. anguivi* compared to all existing groups on days 8 and 12 posttreatment.

### 3.3. Effects of Hydromethanolic Extract *S. anguivi* Fruit Treatment on Packed Cell Volume of *T. congolense* Infected Mice

**Figure 2:**
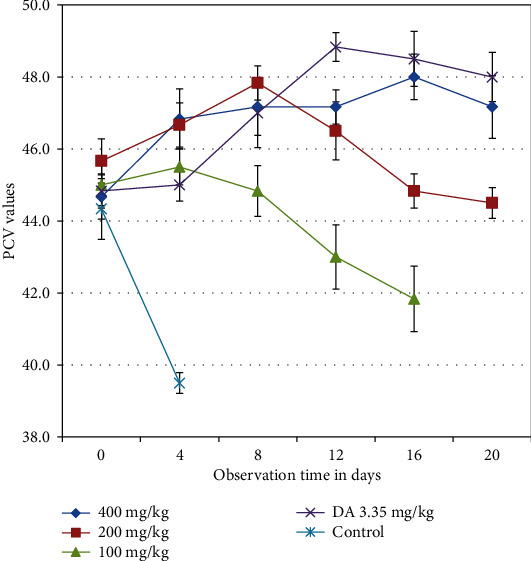
Effect of *S. anguivi* fruit extract treatment on PCV of *T. congolense* infected mice. Values are expressed as mean + SEM; day 0: the 10th day after infected blood inoculation.

Groups of mice treated with 100, 200, and 400 mg/kg of *S. anguivi* and DA3.35 mg/kg showed statistical significance (*p* < 0.05) improvement in PCV measurement on day 4 posttreatment initiation compared with untreated control. Groups of mice treated with 200 and 400 mg/kg of *S. anguivi* and DA3.35 mg/kg showed statistically significant (*p* < 0.05) improvement in PCV measurements on day 12 to day 16 posttreatment initiation compared with groups treated with 100 mg/kg body weight (Figure 2).

### 3.4. Effects of Hydromethanolic Extract of *S. anguivi* Fruit Treatment on Body Weight of *T. congolense* Infected Mice

**Figure 3:**
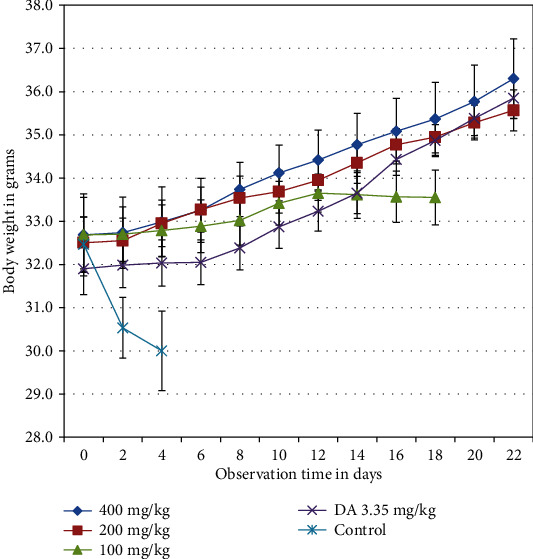
The effect of crude hydromethanolic extracts of *S. anguivi* fruit on body weight of *T. congolense* infected mice. Values are expressed as mean + SEM; day 0: the 10th day after infected blood inoculation.

The mean body weight for *S. anguivi* indicates that there is a gradual increase through day 0-day 12 posttreatment for all treatments with a gradual decrease for untreated control at which all animals in the last group and before day 7 posttreatment. The result indicates that the groups treated with higher doses and DA showed higher mean body weight than that of 100 mg/kg body weight, in which the last group showed a slight decrease in body weight until all animals of the group died at day 18 posttreatment (Figure 3).

### 3.5. Effects of Hydromethanolic Extract of *S. anguivi* Fruit Treatment on Rectal Temperature of T. congolense Infected Mice

**Figure 4:**
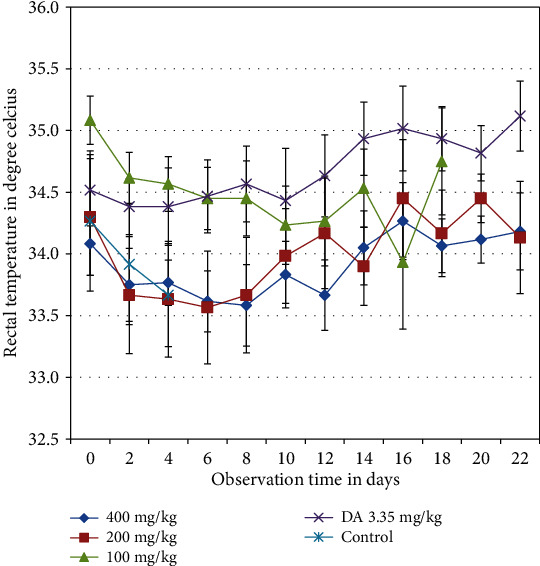
The effect of crude hydromethanolic extracts of *S. anguivi* fruits on rectal temperature of T. congolense infected mice. Values are expressed as mean + SEM; day 0: the 10th day after infected blood inoculation.

Throughout the experiment, the rectal temperatures of the animals fluctuated. In the period of follow-up, no differences have been observed (Figure 4).

### 3.6. Effects of Hydromethanolic Extract of *S. anguivi* Fruit Treatment on the Survival Period of *T. congolense* Infected Mice

Death in vehicle (nontreated mice) started 2 days' posttreatment initiation in all mice in which all mice end at 14.83 ± 0.48 whereas survival time infected mice treated with *S. anguivi* fruits continues 41.83 ± 1.17 days, respectively. Maximum survival period was recorded with DA3.35 mg/kg and *S. anguivi* fruits 400 mg/kg body weight. The mean survival time of all groups treated with extract and DA3.35 mg/kg showed that statistical significance (*p* < 0.05) increases in survival days compared with those not treated (untreated control) where higher dose of the extract and DA treated mice showed statistical significance (*p* < 0.05) increase survival days as compared with those groups treated with 100 mg/kg body weight (Table 1).

### 3.7. Phytochemical Screening

Phytochemical screening showed the presence of various phytochemicals in the extracts. It was found that the extracts had phytochemicals like saponins, tannins, phenols, terpenes, flavonoids, glycosides, alkaloids, anthraquinones, steroids, and flavonoids.

## 4. Discussions

*In vivo* antitrypanosomal effect showed that extract decreased the parasite, increased survival days of extract challenged and treated mice as compared to the untreated controls. The probable reason that the parasite is decreased in the blood could be as a result of access of the extract to the parasites in the blood. Though the extract fails to eliminate parasite from blood of infected mice, it reduced level of parasitaemia [19, 32–34]. The increased survival of *S. anguivi* Lam challenged and treated mice in this study could be related to the action of the active compounds in the extract on red blood cells and/or antioxidant activity.

The current finding is in line with finding of Tadesse et al. [35] who reported that treatment with *D. abyssinica* extract resulted in level of parasitemia and Kifleyohannes [36] who reported animals treated with *A. absinthium* and *M. stenopetala* extracts reduced parasite count approximately half that of untreated control [17], and [23] reported that active ingredients from *A. maciverae* and *A. indica* significantly reduce parasitemia with a dose-dependent effect. The current finding indicates that the extract contains some trypanocidal agent that is active for both chemoprophylaxis and chemotherapeutics of the disease.

The result shows that the extract of the plant has the capacity to improve the PCV even if it may decline after relapse of parasites. The relapsing of parasitemia or in ability of the extract to fully eliminate the parasite is related to low amounts of the active compound in the crude extract and also high virulence of the test organism. The mean PCV between standard drug and different doses of the extract were relatively comparable as the findings of Kifleyohannes et al. and Tadesse et al. [35, 36]. Decrease in mean PCV from untreated controls may be as a result of anemia [19, 37]. The ability to improve PCV is the reason for the long time survival of treated mice. This is possibly by reducing parasite load or in activating the toxic metabolites produced by trypanosome or enhancing resistance of erythrocyte hemolysis [10, 38, 39]. In this study, the results suggest that the ability of extracts to prolong mice's lives could be attributed to their ability to support their antioxidant defense system.

Anemia is a cardinal sign of trypanosome infections. The mechanism of anemia due to this infection is complex and multifactorial in origin. Packed cell volume usually gives an indication of the anemia and status of a trypanosome infected animal [40]. Hepatic injury induced by trypanosomiasis is one of the major health problems to both wild and domestic animals and human beings [7].

The gradual increase in mean body weight for extract treated mice through day 0 to day 12 posttreatment for all treatments with a gradual decrease for untreated control is in agreement with the findings of [41] who reported a slight increase in body weight [8] and who reported treatment with the crude extracts of *Z. officinale* prevented loss of weight associated with parasitaemia [36] also reported that the weight in the untreated infected mice group started to decrease after 12 days' postinfection till the mice died by day 18 where as those on standard drugs and extracts of *A. absinthium* and *M. stenopetala* treated mice generally showed a gradual increase in mean weight until the end of the experimental period. The finding is not in agreement with the reports of [23] who reported that the extract of *A. indica* and suramin-treated groups had a significant decline in body weight.

The extract of *S. anguivi* fruits was found to contain saponins, tannins, phenols, terpenes, flavonoids, glycosides, and alkaloids. Preliminary phytochemical screening of potent plants against trypanosomes showed the presence of these known bioactive compounds in the crude plant extracts tested [42]. The stem, fruits, roots, flowers, and leaves of *S. anguivi* contain alkaloids, solamargine, and solasoline [43].

It has been known that flavonoids and flavonoid-derived plant natural products are effective antitrypanosomal substances against different trypanosome species [44, 45]. Phenolics and polyphenols have been reported in the literature to have antitrypanosomal potential by inhibiting the trypanosome alternative oxidase [46]. Saponins from *S. anguivi* fruits exhibit free radical scavenging activities that possess reducing power, potent antioxidan, and iron chelating ability, making it an excellent candidate in the treatment of diseases in which reactive oxygen species have been implicated [47]. Secondary plant metabolites from *S. anguivi* extract are able to reduce reactive oxygen species generated during infections, and all parameters of the infected mice were maintained during chemoprophylaxis and chemotherapy.

Acute toxicity study revealed that the extract is nontoxic since no treatment-related signs of toxicity were noticed in the animals throughout the observation period at the dose of 2000 mg/kg, which is in agreement with the results of [48, 49]. Garner and Clarke [50] had reported that a substance has low toxicity if its LD50 is 1000 mg/kg body weight. Similarly, Lorke [30] classified substances as slightly toxic if their LD50 ranges from 100 to 1000 mg/kg body weight. According to Garner and coworkers, any compound or drug with an oral LD50 estimate greater than 1,000 mg/kg could be considered low toxic and safe [50]. In previous study by Abdeta et al. [51], it was provided that *E. kebericho* roots have been tested on *T. congolense* infected mice and found effective at crude level with nonobservable toxic level indicating that the claimed ethno medicine was found without acute toxicity at crude level.

## 5. Limitations of the Study

This study was undertaken only on 80% methanol extract of *S. anguivi* and lacks powerful and deep investigation of its pharmacological qualities with their structural activity relationship.

## 6. Conclusions

Studies on the effects of plant extract on Trypanosoma parasite infection are increasing with promising results. In conclusion, the results obtained from this study put evidence that hydromethanolic extract of *Solanum anguivi* Lam fruits possesses antitrypanosomal activity against field isolates of *Trypanosoma congolense*. It also provides evidence for traditional use of the plant for management animal trypanosomiasis in Ethiopia. Since current study was carried out on crude extract of the plant, further investigations are needed to identify and isolate pure compound from the plant and determine its mechanism of action.

## Figures and Tables

**Table 1:** Effects of hydromethanolic extract of *S. anguivi* fruit treatment on mean survival time of *T. congolense* infected mice.

Dose	Mean survival time in days
100 mg/kg	31.83 ± 1.14^a^ (28.91-34.76)
200 mg/kg	39.67 ± .88^a^ (37.40-41.93)
400 mg/kg	41.83 ± 1.17^ac^ (38.83-44.83)
DA3.35 mg/kg	43.00 ± 1.21^ac^ (39.89-46.11)
Control	14.67 ± .88 (12.40-16.93)

Values are expressed as mean ± SEM and 95% CI (values in bracket); the mean difference is significant at the 0.05 level; control: distilled water; DA: diminazine aceturate. ^a^Compared to untreated control; ^c^Compared to 100 mg/kg.

## Data Availability

The authors will provide all data upon request.
